# The role of adjuvant chemotherapy in nasopharyngeal carcinoma with bulky neck lymph nodes in the era of IMRT

**DOI:** 10.18632/oncotarget.7849

**Published:** 2016-03-02

**Authors:** Tingting Xu, Chunying Shen, Xiaomin Ou, Xiayun He, Hongmei Ying, Chaosu Hu

**Affiliations:** ^1^ Department of Radiation Oncology, Fudan University Shanghai Cancer Center, Shanghai, China; ^2^ Department of Oncology, Shanghai Medical College, Shanghai, China

**Keywords:** nasopharyngeal carcinoma, locally advanced, N2–3, intensity modulated radiation therapy, adjuvant chemotherapy

## Abstract

Nasopharyngeal carcinoma (NPC) patients with N2–3 diseases are prone to develop distant metastasis even treated with standard concurrent chemoradiotherapy (CCRT). Our study is aim to determine the optimal treatment strategy of these patients. Patients with histologically proven NPC were retrospectively analyzed according to the AJCC 2002 stage classification system. A total of 547 patients who had N2–3 diseases were enrolled. They were all treated with Intensity-modulated radiation therapy (IMRT) combined with systemic treatments, including radiotherapy alone (RT alone), neoadjuvant chemotherapy followed by radiotherapy (NACT+RT), CCRT, NACT+CCRT, NACT followed by radiotherapy and adjuvant chemotherapy (NACT+RT+AC), CCRT+AC and NACT+CCRT+AC. A subgroup analysis was also conducted. With a median follow-up time of 53.8 months, adjuvant chemotherapy significantly decreased the risk of distant metastasis (HR 0.413, 95% CI 0.194–0.881, *p* = 0.022) as well as significantly increased the OS (HR 0.398, 95% CI 0.187–0.848, *p* = 0.017) in patients with N3 disease. The addition of adjuvant chemotherapy seemed to provide benefits to patients with N3 stage NPC and the current study may indicate the need for further randomized investigation.

## INTRODUCTION

Platinum-based concurrent chemoradiotherapy represents the gold standard in the treatment of locally advanced nasopharyngeal carcinoma (NPC) [1, 2] in the era of 2D radiotherapy. Intensity-modulated radiation therapy (IMRT) has greatly improved the local control rate to above 90% [3] but failed to further reduce the distant metastases of patients with bulky lymph nodes (N2–3) [4]. Approximately up to 30–40% patients with of N3 stage will develop distant failures after radical treatment [3, 5]. Therefore, it is crucial to choose proper chemotherapeutic modality to maximally reduce distant invasion.

There are limited data of randomized clinical trials with adjuvant chemotherapy to support evidence-based decision-making [6–8]. All studies had failed to demonstrate significant advantage in whole population of locally advanced disease without risk stratification.

On the basis of this background, we retrospectively explored the possible treatment option and hypothesized that patients with bulky lymph nodes would benefit from additional chemotherapy other than the concurrent modality.

## RESULTS

### Patient characteristics and treatment modalities

According to the systemic treatment modalities delivered, we classified all 547 patients into non-adjuvant chemotherapy (non-AC) group (341 patients) and adjuvant chemotherapy (AC) group (206 patients). All characteristics except AJCC staging of them were balanced across the two treatment groups in N2–3 population, more stage IVb (N3) patients were prone to receive AC (*P =* 0.005). In the subgroup analyses of 407 N2 and 140 N3 individuals, with 269 non-AC/138 AC patients and 72 non-AC/68 AC patients included, respectively, all of them had well-balanced characteristics (Table 1A–1B). Table 2A–2B listed all the treatment modalities administered in those patients. Regimens and cycles of chemotherapy delivered had also been particularized in detail. Among the whole population, NACT+CCRT and NACT+RT+AC were the most frequently used regimen in the non-AC group (43.0%) and AC group (33.5%), respectively, and likewise in the N2 (45.0% and 30.5%) and N3 subgroup (37.1% and 42.2%). The cycles of neoadjuvant and concurrent chemotherapy received in different treatment groups were shown in Table 3, and except for the AC, differences of other treatments were not significant.

**Table 1A:** Characteristics of 547 N2–3 NPC patients stratified by adjuvant chemotherapy

	N2–3 (*n* = 547)		
	non-AC group (%) (*n* = 341)	AC group (%) (*n* = 206)	*P*
Age			0.097
Median (yrs)	46	48	
Range (yrs)	7–75	15–70	
Sex			0.660
Male	254 (74.5)	158 (76.7)	
Female	87 (25.5)	48 (23.3)	
KPS score			0.294
80–100	338 (99.1)	206 (100.0)	
60–70	3 (0.9)	0 (0.0)	
T stage			0.204
T1	79 (23.2)	40 (19.4)	
T2A/2B	134 (39.3)	91 (44.2)	
T3	95 (27.8)	47 (22.8)	
T4	33 (9.7)	28 (13.6)	
AJCC stage			0.005*
III	240 (70.4)	118 (57.3)	
IVa	29 (8.5)	20 (9.7)	
IVb	72 (21.1)	68 (33.0)	
RT dose			0.305
Median (Gy)	66	66	
Range (Gy)	63.8–77.3	60–74.8	

* indicated *p* < 0.05.

**Table 1B:** Characteristics of N2 and N3 subgroup patients stratified by adjuvant chemotherapy

	N2 (*n* = 407)			N3 (*n* = 140)		
	non-AC group (%) (*n* = 269)	AC group (%) (*n* = 138)	*P*	non-AC group (%) (*n* = 72)	AC group (%) (*n* = 68)	*P*
Age			0.570			0.080
Median (yrs)	46	47.5		46	51	
Range (yrs)	11–75	15–70		7–74	16–68	
Sex			0.386			0.106
Male	204 (75.8)	102 (73.9)		50 (69.4)	56 (82.4)	
Female	65 (24.2)	36 (26.1)		22 (30.6)	12 (17.6)	
KPS score			0.554			1.000
80–100	266 (98.9)	138 (100.0)		72 (100.0)	68 (100.0)	
60–70	3 (1.1)	0 (0.0)		0 (0.0)	0 (0.0)	
T stage			0.684			0.252
T1	57 (21.2)	27 (19.6)		22 (30.5)	13 (19.1)	
T2A/2B	105 (39.0)	58 (42.0)		29 (40.3)	33 (48.5)	
T3	78 (29.0)	33 (23.9)		17 (23.6)	14 (20.6)	
T4	29 (10.8)	20 (14.5)		4 (5.6)	8 (11.8)	
AJCC stage			0.334			1.000
III	240 (89.2)	118 (85.5)		0 (0.0)	0 (0.0)	
IVa	29 (10.8)	20 (14.5)		0 (0.0)	0 (0.0)	
IVb	0 (0.0)	0 (0.0)		72 (100.0)	68 (100.0)	
RT dose			0.848			0.280
Median (Gy)	66	66		66	66	
Range (Gy)	63.8–74.4	60–74.8		66–77.3	62.8–70.4	

**Table 2A:** Treatment regimens of 547 N2–3 NPC patients stratified by adjuvant chemotherapy

	N2–3 (*n* = 547)	
	non-AC group (%) (*n* = 341)	AC group (%) (*n* = 206)
Treatment modality		
RT alone	14 (2.6)	0
NACT+RT	65 (11.9)	0
CCRT	27 (4.9)	0
NACT +CCRT	235 (43.0)	0
CCRT+AC	0	3 (0.5)
NACT+RT+AC	0	183 (33.5)
NACT+CCRT+AC	0	20 (3.6)
CT Regimen		
PF	N/A	62 (30.1)
TPF	N/A	86 (41.7)
GP	N/A	58 (28.2)
AC cycles		
1 cycle	N/A	82 (39.8)
2 cycles	N/A	63 (30.6)
3 cycles	N/A	61 (29.6)

Abbreviations: RT = radiotherapy; NACT = neoadjuvant chemotherapy; CCRT = concurrent chemoradiotherapy; AC = adjuvant chemotherapy; CT = chemotherapy.

**Table 2B:** Treatment regimens of N2 and N3 subgroup patients stratified by adjuvant chemotherapy

	N2 (*n* = 407)		N3 (*n* = 140)	
	non-AC group (%) (*n* = 269)	AC group (%) (*n* = 138)	non-AC group (%) (*n* = 72)	AC group (%) (*n* = 68)
Treatment modality				
RT alone	13 (3.2)	0	1 (0.7)	0
NACT+RT	50 (12.3)	0	15 (10.7)	0
CCRT	23 (5.7)	0	4 (2.9)	0
NACT +CCRT	183 (45.0)	0	52 (37.1)	0
CCRT+AC	0	2 (0.5)	0	1 (0.7)
NACT+RT+AC	0	124 (30.5)	0	59 (42.2)
NACT+CCRT+AC	0	12 (2.9)	0	8 (5.7)
CT Regimen				
PF	N/A	57 (41.3)	N/A	29 (42.7)
TPF	N/A	49 (35.5)	N/A	13 (19.1)
GP	N/A	32 (23.2)	N/A	26 (38.2)
AC cycles				
1 cycle	N/A	54 (39.1)	N/A	28 (41.2)
2 cycles	N/A	37 (26.8)	N/A	26 (38.2)
3 cycles	N/A	47 (34.1)	N/A	14 (20.6)

Abbreviations: RT = radiotherapy; NACT = neoadjuvant chemotherapy; CCRT = concurrent chemoradiotherapy; AC = adjuvant chemotherapy; CT = chemotherapy.

**Table 3:** The cycles of NACT and CCT received in different treatment groups

	Non-AC group (%)	AC group (%)	
	NACT+RT	NACT+RT+AC	*P* value
NACT cycles 1	4 (6.2)	8 (4.4)	0.742
2	42 (64.6)	114 (62.3)	
3	19 (29.2)	61 (33.3)	
	CCRT	CCRT+AC	*P* value
CCT cycles < 5	16 (59.3)	1 (33.3)	0.565
≥ 5	11 (40.7)	2 (66.7)	
	NACT+CCRT	NACT+CCRT+AC	*P* value
NACT cycles 1	4 (1.7)	0 (0.0)	0.384
2	177 (75.3)	13 (65.0)	
3	54 (23.0)	7 (35.0)	
CCT cycles < 5	132 (56.2)	9 (45.0)	0.358
≥ 5	103 (43.8)	11 (55.0)	

Abbreviations: NACT = neoadjuvant chemotherapy; CCRT = concurrent chemoradiotherapy; CCT = concurrent chemotherapy; AC = adjuvant chemotherapy.

### Treatment failures

Sites of locoregional relapse and distant metastases for the non-AC and AC groups were shown in the Table 4A–4B. We compared the failure patterns and discovered no significant differences between non-AC and AC groups in all N2–3 patients (*P =* 0.859 for relapse and *P =* 0.345 for metastasis). Nevertheless, AC was found to reduce the distant metastasis rate significantly in N3 subgroup (*P =* 0.013). Similar relapse rates were found (9.7% and 14.7%, *P =* 0.610) but still more patients progressed in the non-AC group (28/72(38.9%) vs 23/68(33.8%), *P =* 0.534). Among them, 7/17 (41.2%) relapsed patients and 24/37 (64.9%) metastatic patients died before the last follow-up, respectively.

**Table 4A:** Treatment failure patterns of 547 N2–3 NPC patients stratified by adjuvant chemotherapy

	N2–3 (*n* = 547)		
	non-AC group (%) (*n* = 341)	AC group (%) (*n* = 206)	*P*
Relapses			0.859
Nasopharynx	12 (3.5)	5 (2.4)	
Base of skull	6 (1.8)	4 (2.0)	
Neck	18 (5.3)	8 (3.9)	
Naso+neck	10 (2.9)	5 (2.4)	
Total	46 (13.5)	22 (10.7)	
No relapse	295 (86.5)	184 (89.3)	
Metastases			0.345
Liver	14 (4.1)	3 (1.5)	
Bone	29 (8.5)	15 (7.3)	
Lung	13 (3.8)	13 (6.3)	
Other	5 (1.5)	2 (0.9)	
Multiple	12 (3.5)	6 (2.9)	
Total	73 (21.4)	39 (18.9)	
No metastasis	268 (78.6)	167 (81.1)	

**Table 4B:** Treatment failure patterns of N2 and N3 subgroup patients stratified by adjuvant chemotherapy

	N2 (*n* = 407)			N3 (*n* = 140)		
	non-AC group (%) (*n* = 269)	AC group (%) (*n* = 138)	*P*	non-AC group (%) (*n* = 72)	AC group (%) (*n* = 68)	*P*
Relapses			0.114			0.610
Nasopharynx	11 (4.1)	3 (2.2)		1 (1.4)	2 (2.9)	
Base of skull	5 (1.8)	3 (2.2)		1 (1.4)	1 (1.5)	
Neck	14 (5.2)	5 (3.6)		4 (5.5)	3 (4.4)	
Naso+neck	9 (3.3)	1 (0.7)		1 (1.4)	4 (5.9)	
Total	39 (14.5)	12 (8.7)		7 (9.7)	10 (14.7)	
No relapse	230 (85.5)	126 (91.3)		65 (90.3)	58 (85.3)	
Metastases			0.893			0.013*
Liver	11 (4.1)	3 (2.2)		3 (4.1)	0 (0.0)	
Bone	14 (5.2)	9 (6.5)		15 (20.8)	6 (8.8)	
Lung	10 (3.7)	11 (8.0)		3 (4.2)	2 (2.9)	
Other	3 (1.1)	2 (1.4)		2 (2.8)	0 (0.0)	
Multiple	11 (4.1)	1 (0.7)		1 (1.4)	5 (7.4)	
Total	49 (18.2)	26 (18.8)		24 (33.3)	13 (19.1)	
No metastasis	220 (81.8)	112 (81.2)		48 (66.7)	55 (80.9)	

* indicated *p* < 0.05.

### Survivals

**Figure 1:**
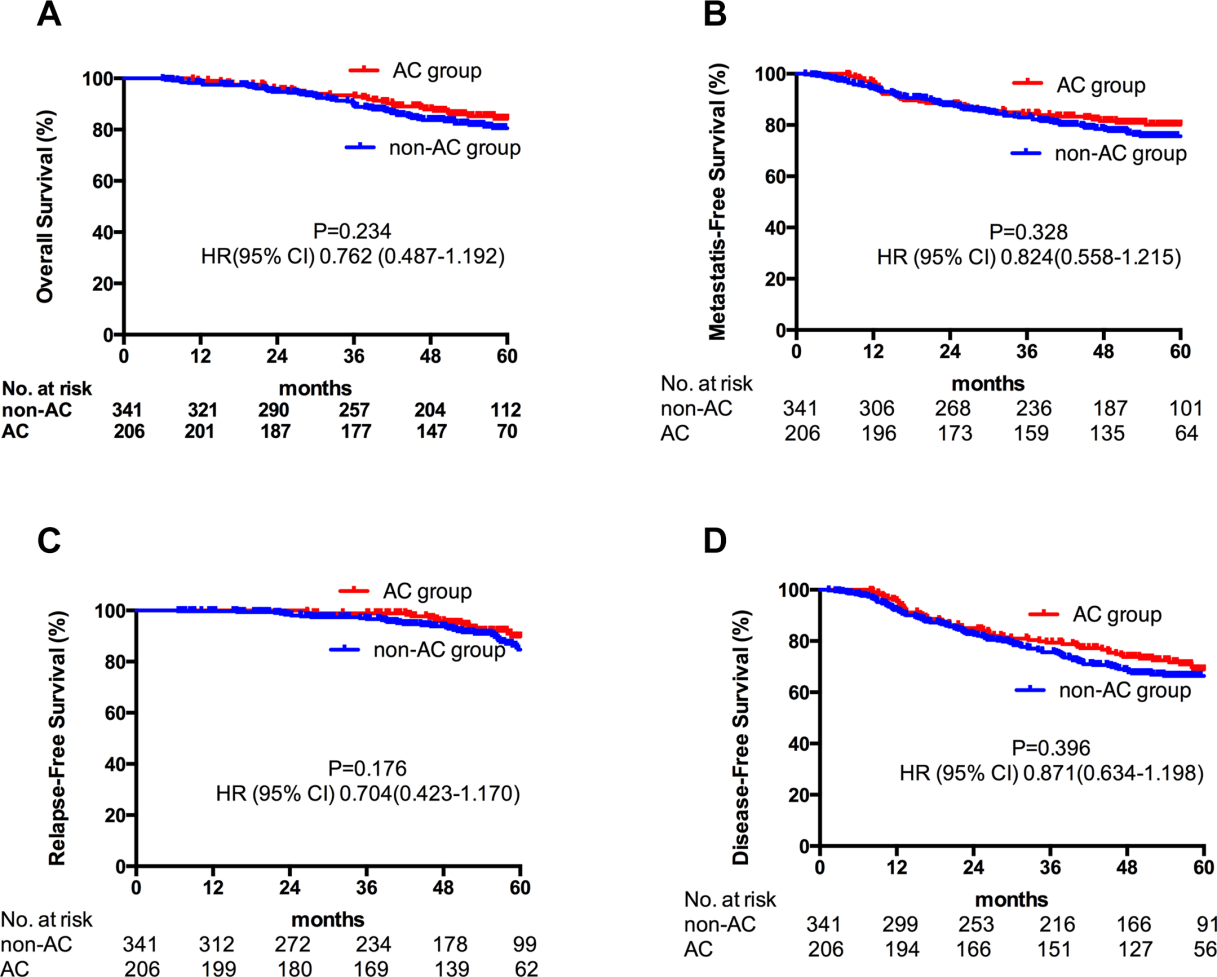
Kaplan-Meier estimate of OS (A), DMFS (B), LRFS (C) and DFS (D) in all N2–3 patients stratified by adjuvant chemotherapy

**Figure 2:**
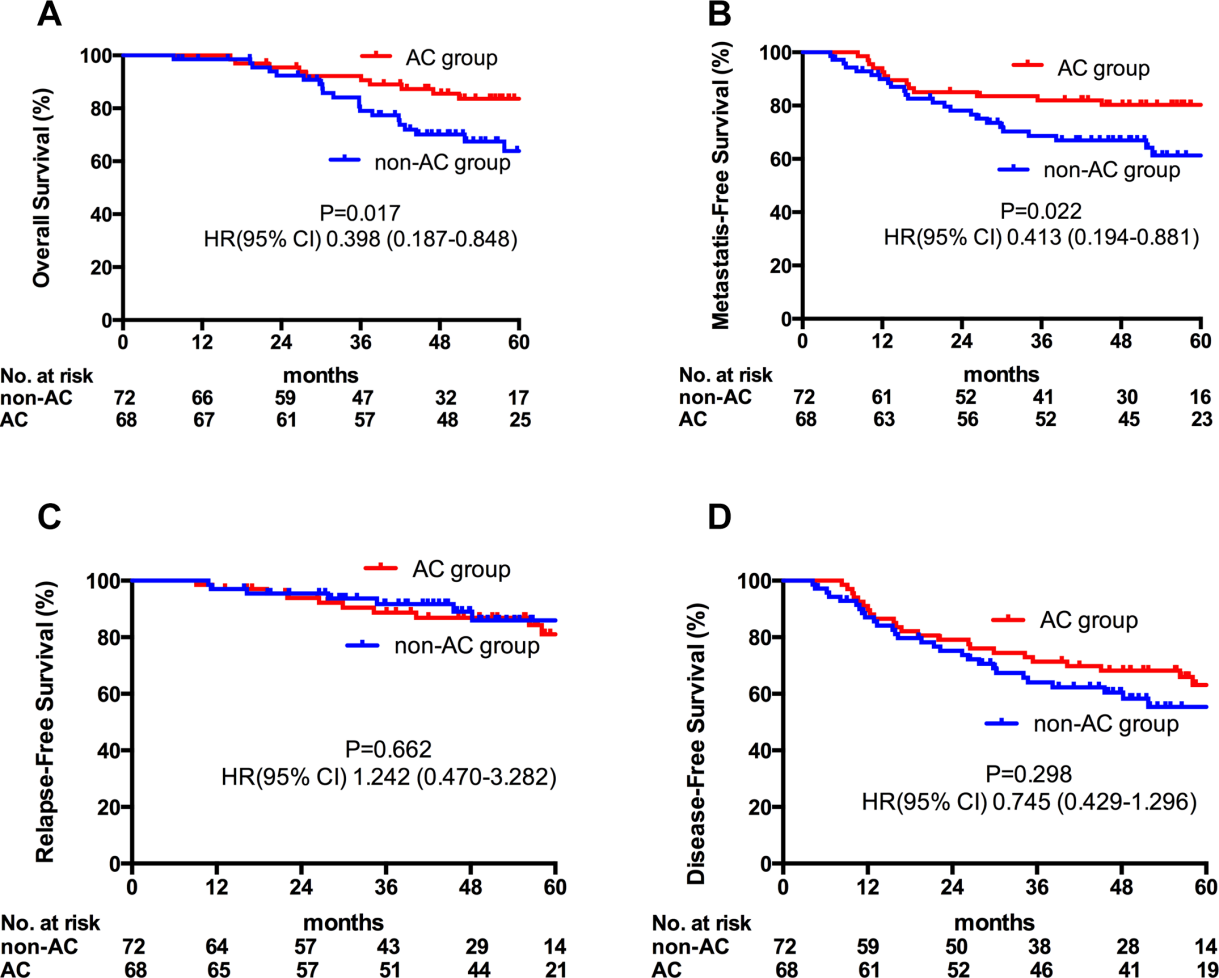
Kaplan-meier estimate of OS (A), DMFS (B), LRFS (C) and DFS (D) in N3 patients stratified by adjuvant chemotherapy

With a median follow-up time of 53.8 (3.0–79.1) months, AC added no further benefits to all N2–3 NPC patients (Figure 1A–1D). However, in subgroup analyses, the addition of AC significantly decreased the risk of distant metastasis (HR 0.413, 95% CI 0.194–0.881, *p =* 0.022) as well as significantly increased the OS (HR 0.398, 95% CI 0.187–0.848, *p =* 0.017) in patients with N3 diseases (Figure 2A–2D). The effect of adjuvant chemotherapy was especially noteworthy for reduction in distant metastatic events, rather than for local or regional recurrences in patients with lymph nodes larger than 6cm (N3a) and/or supraclavicular fossa invasion (N3b).

In a further exploratory analysis, different chemotherapy regimens also engendered different consequences (Table 5). TPF and GP were demonstrated to have a trend to be superior to PF in improving OS, but the difference not significant (89.3% and 86.8% vs 71.6%, *P =* 0.157). The chemotherapy cycles delivered were not associated with the outcomes, either.

**Table 5:** Relationships between the regimens/cycles of adjuvant chemotherapy and survival outcomes in N3 population

	Regimens	Survival rates	*P*	Cycles	Survival rates	*P*
4y OS	TPF	89.3%	0.157	1	85.2%	0.580
	PF	71.6%		2	86.9%	
	GP	86.8%		3	81.8%	
4y DMFS	TPF	79.3%	0.592	1	82.1%	0.892
	PF	91.7%		2	76.5%	
	GP	76.5%		3	84.6%	
4y LRFS	TPF	92.7%	0.162	1	88.2%	0.245
	PF	71.4%		2	87.8%	
	GP	86.5%		3	80.8%	
4y DFS	TPF	72.4%	0.832	1	71.4%	0.594
	PF	64.3%		2	65.2%	
	GP	64.6%		3	65.9%	

Abbreviations: OS = Overall Survival; DMFS = Distant Metastasis-Free Survival; LRFS = Locoregional Relapse-Free Survival; DFS = Disease-Free Survival.

## DISCUSSION

The clinical advantages of IMRT in the treatment of NPC with respect to both disease control and adverse-effect profiles have been repeated demonstrated [9–14]. It was not difficult to understand that the benefit of locoregional control derived from concurrent chemotherapy would be weakened by the modern radiotherapy technique (IMRT). Distant metastasis became the main treatment failure pattern in locally advanced NPC especially with advanced N stage.

It is important to realize that patients with bulky neck lymph nodes (especially N3) are a heterogeneous group, and thus the strategies for treatment should differ depending on the lymph nodal status of the individual patient. Several studies had demonstrated that N3 patients would have more chances to develop metastases hence achieved poorer overall survival rates. Cheng et al. [15] found the 3-year DMFS rate were 92%, 84% and 56% (*P =* 0.003) in N0–1, N2 and N3 subgroup, respectively. T4 and N3 were discovered to be two independent prognostic factors to influent distant metastases in further Cox regression analysis. This is consistent with Liu's series [16] in which the 3-year OS rate of N1–2 was 85% and sharply down to 58% in N3 patients (*P =* 0.046).

Despite being the NCCN recommendation for locally advanced NPC, CCRT may not always be the only choice. In Lin's study [17], patients were divided into different metastatic risk subgroups by nodal status and tumor stage. CCRT was proved to be superior to RT alone for low-risk patients (83.2% vs 59.7% for OS at 5-year *P =* 0.004) but inadequate for high-risk ones (55.8% vs 46.3% for OS at 5-year *P =* 0.176). It is reasonable to presume that an alternative modality should be further explored for advanced N patients.

The question remains unclear whether increasing chemotherapy intensity can improve the outcome of those patients. Several randomized studies [6–8] have attempted to identify the effectiveness of adjuvant chemotherapy in locally advanced diseases (stage III–IVb) and failed to get the expected outcomes. It's a pity that none of them had focused on this specific subgroup. The deficiency of the most published works was that they treated patients without stratification.

**Figure 3:**
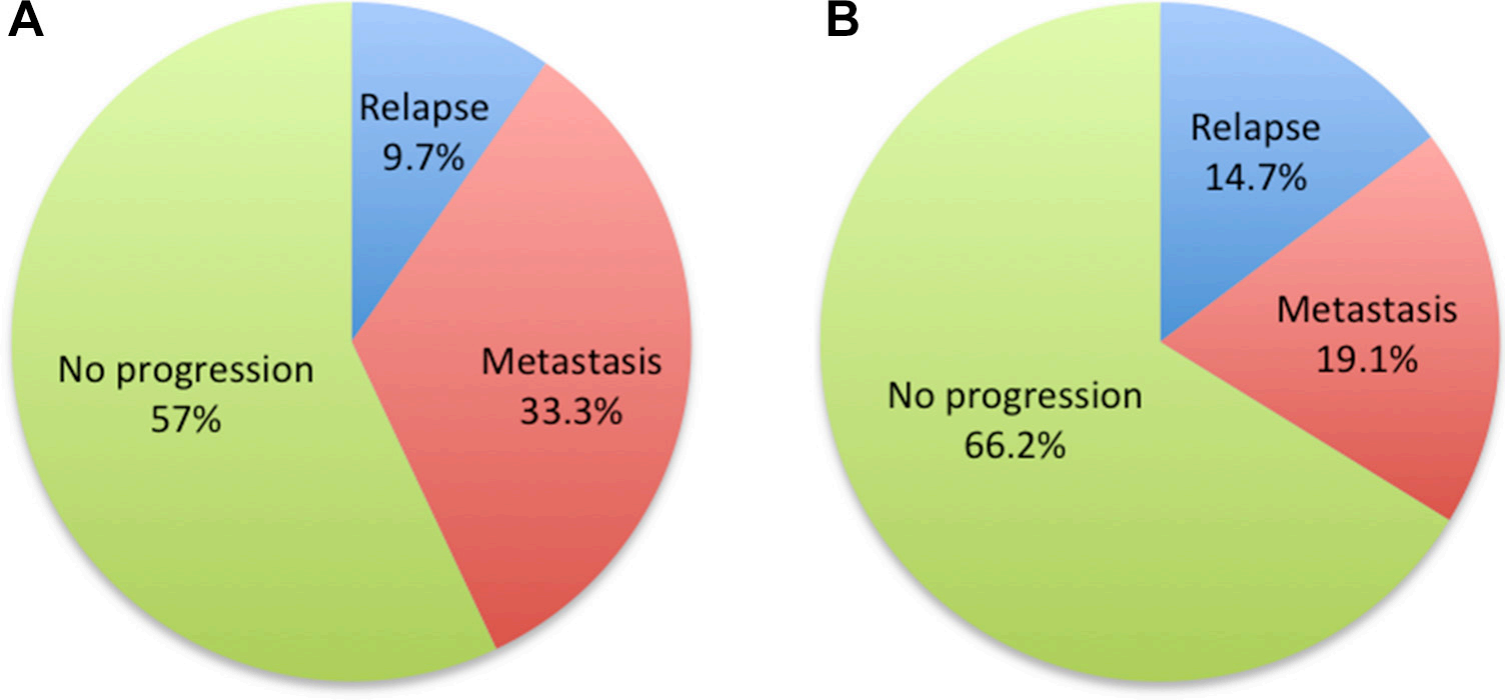
Pie charts of treatment failure patterns of non-AC (A) and AC (B) groups

We were first to report the achievements by different lymph nodal status. In this retrospective study, the addition of adjuvant chemotherapy seemed to provide delightful benefit to patients with N3 stage NPC. The overall survival was largely improved in terms of the reduced distant metastasis. In the analyses of failure patterns, similar relapse rates (9.7% vs 14.7%, *P =* 0.610) and more metastasis rates (33.3% vs 19.1%, *P =* 0.013) were shown in the non-AC group (Figure 3A–3B). Among these disease progressions, more metastatic patients (64.9% vs 41.2%) died before the last follow-up. As a result, the differences were still significant in terms of OS. It could be explained by that patients who developed metastases had lost the limitation of diseases and would have more complications thus resulted in shorter survival times. We postulated that metastasis rather than relapse was the determined factor of overall survival. The key of improving outcomes of NPC patients with bulky lymph nodes should be finding a way to reduce the distant metastasis. Systemic chemotherapy is an effective treatment for diminishing micro-metastases that cannot be detected by regular imagining with increased side effects. In the current cohort, AC had showed promisingly survival benefits for N3 patients, indicating delivering chemotherapy after the radical radiotherapy might further decrease the risk of distant metastases.

It had been discovered as well that although TPF and GP were demonstrated to have a trend to be superior to PF in improving OS, the *P* value was still not significant due to the relatively small sample size. As already confirmed by randomized trails [18, 19] and meta-analysis [20] in head and neck squamous cell carcinoma (HNSCC), the TPF regimen is accepted to be a better choice than PF in HNSCC patients who received NACT. Nevertheless, the conclusion is still pending in NPC, randomized investigation are warranted. Moreover, It's not easy to balance the efficacies and toxicities when treating patients, and we must realize that it's also an important affair to recognize predictive factors to select patients who may really have greater benefit from adjuvant therapy.

## MATERIALS AND METHODS

### Patient selection

Between November 2008 and November 2011, a total of 547 any T (T1–4), N2–3, without metastatic disease (M0), NPC patients (according to the AJCC 2002 stage classification system) treated with IMRT-based systemic therapy at Fudan University Shanghai Cancer Center were retrospectively enrolled. Pretreatment evaluation consisted of complete history and physical examination, complete blood cell count, biochemical profile, contrasted-MRI of the nasopharynx and neck, chest CT, ultrasound of the abdomen, bone scan and/or PET/CT.

### Treatment

All 547 patients underwent the same radical radiotherapy (IMRT) and different systemic treatments including radiotherapy alone (RT alone), neoadjuvant chemotherapy followed by radiotherapy (NACT+RT), concurrent chemoradiotherapy (CCRT), neoadjuvant chemotherapy followed by concurrent chemoradiotherapy (NACT+CCRT), concurrent chemoradiotherapy followed by adjuvant chemotherapy (CCRT+AC), neoadjuvant chemotherapy followed by radiotherapy and adjuvant chemotherapy (NACT+RT+AC), neoadjuvant chemotherapy followed by concurrent chemoradiotherapy and adjuvant chemotherapy (NACT+CCRT+AC). Treatment strategy of each patient was made by his/her radiation oncologist.

The IMRT treatment plans were designed and optimized using an inverse planning system (Pinnacle 3, Philips). 6-MV photons were used to treat primary tumors and neck drainage areas. The gross tumor volume (GTV) was delineated based on the findings of MRI. The clinical target volume (CTV) was delineated with adequate margins surrounding the GTV considering the anatomic boundary of the possibly involved subsites. The prescription doses were 66Gy/30Fx and 70.4Gy/32Fx to T1–2 and T3–4 primary diseases, respectively. All metastatic lymph nodes detected on MRI were treated with a prescription dose of 66Gy.

In patients received CCRT, cisplatin was delivered weekly at a dose of 40 mg/m^2^ concurrently with radiotherapy.

In patients received neoadjuvant/adjuvant chemotherapy, the choice of chemotherapy regimen was left to the discretion of the treating medical oncologist. Generally, one of three cisplatin-based neoadjuvant/adjuvant regimens for 1–3 cycles were delivered: PF (DDP 75 mg/m^2^ d1+5-FU 500 mg/m^2^/d with 120-h infusion), TPF (docetaxel 75 mg/m^2^ d1+DDP 75 mg/m^2^ d1–3+5-FU 500 mg/m^2^/d with 120-h infusion) or GP (gemcitabine 1.0g/m^2^ d1, d8+DDP 75 mg/m^2^ d1). The regimens were repeated every 21 days for neoadjuvant phase and every 4 weeks for adjuvant phase. A total of 2–3 cycles of NACT, 6–7 cycles of concurrent chemotherapy and 2 cycles of AC were assigned in each modality. Patients who had residual disease after finished the planned treatment would receive the third cycle of AC if tolerable.

Follow-up data were to be collected every 3 months through the first two years, every 6 months during years 3–5 and every 12 months in years 6–10 for all patients.

### Endpoints and statistical analysis

The Kaplan-Meier model was used to estimate the survival. Differences in survivals between subgroups were compared with log-rank test. The primary end point of the study was the overall survival (OS). In the analysis of OS, a patient was considered to have death if he dead as a result of any cause. Other outcomes of interest included distant metastasis-free survival (DMFS), locoregional relapse-free survival (LRFS) and disease-free survival (DFS). DFS was defined as the time from diagnosis confirmed to relapsed/metastasized. LRFS and DMFS were defined as the duration from the date of diagnosis confirmed to locoregional relapse and distant metastasis, respectively. The χ^2^ test was used to detect statistical differences in proportions. All statistical analyses were performed using SPSS software (version 20.0). A two-sided ***p*** value < 0.05 was considered to be significant. The survival curves were completed using GraphPad Prism 6.0 software.
